# Molecular Factors and Mechanisms Driving Multidrug Resistance in Uropathogenic *Escherichia coli*—An Update

**DOI:** 10.3390/genes13081397

**Published:** 2022-08-06

**Authors:** Marcin Rozwadowski, Damian Gawel

**Affiliations:** 1 Department of Pediatrics, Bielanski Hospital, Ceglowska 80, 01-809 Warsaw, Poland; 2 Department of Cell Biology and Immunology, Centre of Postgraduate Medical Education, 01-813 Warsaw, Poland

**Keywords:** uroptahogenic *E. coli*, multidrug-resistance, mobile genetic elements, SOS stress response system

## Abstract

The rapid emergence of multidrug-resistant (MDR) bacteria indisputably constitutes a major global health problem. Pathogenic *Escherichia coli* are listed among the most critical group of bacteria that require fast development of new antibiotics and innovative treatment strategies. Among harmful extraintestinal *Enterobacteriaceae* strains, uropathogenic *E. coli* (UPEC) pose a significant health threat. UPEC are considered the major causative factor of urinary tract infection (UTI), the second-most commonly diagnosed infectious disease in humans worldwide. UTI treatment places a substantial financial burden on healthcare systems. Most importantly, the misuse of antibiotics during treatment has caused selection of strains with the ability to acquire MDR via miscellaneous mechanisms resulting in gaining resistance against many commonly prescribed antibiotics like ampicillin, gentamicin, cotrimoxazole and quinolones. Mobile genetic elements (MGEs) such as transposons, integrons and conjugative plasmids are the major drivers in spreading resistance genes in UPEC. The co-occurrence of various bacterial evasion strategies involving MGEs and the SOS stress response system requires further research and can potentially lead to the discovery of new, much-awaited therapeutic targets. Here, we analyzed and summarized recent discoveries regarding the role, mechanisms, and perspectives of MDR in the pathogenicity of UPEC.

## 1. Introduction

More than 130 years ago, the first description of *Escherichia coli* was delivered by Austrian pediatrician Theodor Escherich. The object of his discovery still remains a subject of interest for scientists and clinicians. *E. coli* has earned the status of a classic experimental model organism. Commensal *E. coli* strains constitute one of the cornerstones of the gut microbiome, while some of the pathogenic *E. coli* pose a significant threat to human health [1,2,3].

*E. coli* is a Gram-negative, facultative anaerobic, rod-shaped bacterium. Over 160 serological types of *E. coli* have been classified based on the major surface antigens: O (polymer of oligosaccharides), K (capsular polysaccharides), and H (flagella components) [4,5]. The long-chain O-antigen of bacterial lipopolysaccharide (LPS) presents great structural variability between strains. Until now, over 180 distinct “ohne Hauch” O-antigens, which demonstrate nonspreading growth on agar have been described in *E. coli.* Likewise*,* over 80 separate types of “Kapsel” (K) capsular polysaccharides creating a mucous-like layer surrounding some *E.coli* and 50 types of “Hauch” (H) flagellum antigens, major constituents of flagella, have been identified [5,6,7].

**Figure 1:**
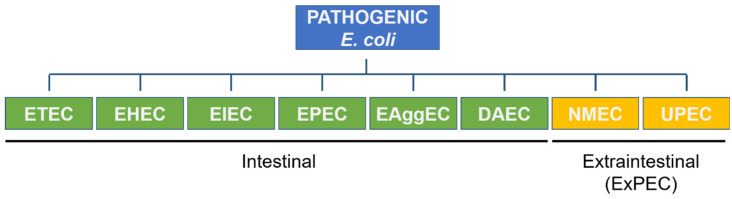
Pathogenic subtypes of *E. coli*. ETEC—Enterotoxigenic *E. coli*; EHEC—Enterohaemorrhagic *E. coli*; EIEC—Enteroinvasive *E. coli*; EPEC—Enteropathogenic *E. coli*; EAggEC—Enteroaggregative *E. coli*; DAEC—Diffusely Adherent *E. coli*; NMEC—Neonatal Meningitis *E. coli*; UPEC—Uropathogenic *E. coli*.

Another classification of *E. coli* is based on the presence of pathogenicity islands in the genome and according to it, *E. coli* can be divided into A, B1, B2, C, D, E, and F groups, and clade I. Non-harmful *E. coli* strains are predominantly designated to the A or B1 group, while pathogenic *E. coli* causing extraintestinal infections are classified into groups B2 and D. Groups E and F are related to the main groups B2 and D, respectively. Interestingly, *E. coli* strains that present an indistinguishable phenotype, but are not genetically identical, are assigned to the cryptic clade I group [7]. Additionally, based on acquired virulence factors and the type of the caused disease, pathogenic *E. coli* can be classified into eight pathotypes, six of which are intestinal and two are extraintestinal (Figure 1) [5,7,8].

Among extraintestinal pathogenic *E. coli* (ExPEC), uropathogenic *E. coli* (UPEC) strains pose a significant health threat, as they cause one of the most common bacterial-born infection in humans—urinary tract infections (UTIs). Treating UTIs is becoming more and more challenging due to increasing resistance of UPEC strains to commonly used antimicrobial agents [9,10].

## 2. Urinary Tract Infections and Uroptahogenic *E. coli*

### 2.1. Epidemiology and Burden of UTI

Every year over 150 million people are diagnosed with UTIs, making them the second most common bacterial infection [11]. The number of cases is certainly underestimated as UTIs are non-reportable diseases in many countries [12]. It is predicted that 50–60% of adult women will develop a UTI at least once in their lifetime [13]. Other studies suggest that the frequency of UTIs in young women is about 0.5 episodes per person, yearly, of which 27% is recurrent, making the number even higher [14,15].

UPEC is the main etiological factor of UTIs and it is responsible for approximately 80–90% of all cases. According to published data, 81% of UPEC strains isolated from patients belong to groups B2 and D [10,11,16,17]. Both groups are characterized by higher distribution of the *fyuA*, *pap*, and *yfcV* genes encoding the ferric yersiniabactin uptake receptor, pyelonephritis-associated pili, and fimbrial-like protein, respectively. These virulence factors take part in iron uptake and cell adhesion [18]. Other virulence genes such as *sfa* (S fimbria), *cnf* (an operon encoding for cytotoxic necrotizing factor type I—CNF-1), and *pic* (factor PIC) were only found within the B2 phylogroup [18]. This is consistent with the recent report by Dadi et al. who also highlighted a strong association between the B2 group and the virulence genes listed above. However, at the same time, the prevalence of the B2 and D groups was reported to be slightly lower than the data presented above and accounted for 57.5% of all UPEC isolates [19].

In the face of the spread of multi-drug resistant (MDR) bacteria and the lack of alternative effective treatment methods, management of recurrent UTIs poses a significant challenge. Treatment of kidney-transplant patients, as well as life-threatening manifestations of ExPEC infections (e.g., sepsis or meningitis) are still associated with high mortality [20,21]. 

Therefore, curing UTIs is also associated with a substantial economic consequence. Medical consultations for UTIs constitute 0.9–6% of all outpatient visits and absences from work, summing up to $3.5 billion per year, in the United States alone [11,15,22].

### 2.2. Pathomechanism of UTI

**Figure 2:**
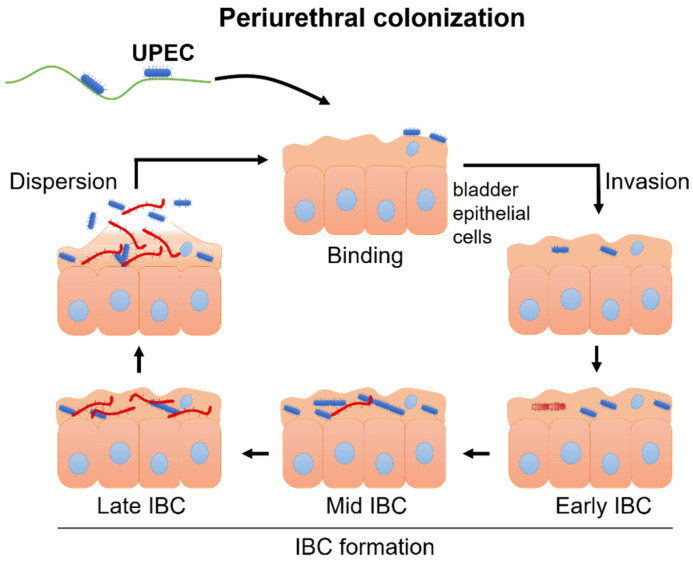
Model of the urinary tract infection cascade. UPEC—uropathogenic *E. coli*; IBC—intracellular bacterial communities.

The general mechanism of the UTI cascade is relatively well understood. An infection begins when UPEC enter through the urethra and reach the epithelial cells of the bladder. Next, bacteria bind to the surface epithelium with use of various adhesins and then the invasion step occurs. As bacteria enter the cytoplasm of the urinary bladder epithelial cells, they actively replicate and form biofilm-like intracellular bacterial communities (IBCs), which hinder an effective immune response and enables further UPEC cell divisions. Lastly, dispersion of uroepithelial cells closes the cycle and the freed UPEC bugs can renew the UTI process (Figure 2) [11,23]. 

Understanding the infection mechanisms has led to the discovery of potential targets in UPEC therapy. For example, three types of fimbriae—Type 1, P, and Curli—are prerequisites for development of UTIs. These virulence factors are critical during uroepithelial cell invasion and acute pyelonephritis, and are associated with adhesion and the IBC formation capabilities of UPEC [24]. In a recent report, Yang et al. showed that administration of dictamine, a plant alkaloid, significantly impairs UPEC’s virulence via downregulation of the expression of fimbriae-encoding genes. This makes dictamine a promising non-antibiotic drug in treatment of UTIs [25]. Nevertheless, the general therapeutic options are limited as antibiotic therapy remains the mainstay of UTI treatment with other non-antibiotic measures such as cranberry products or probiotics playing a supporting role, although of uncertain effectiveness [26,27,28,29].

## 3. Multi-Drug Resistance and UPEC

Increasing antibiotic resistance of bacteria strains observed worldwide remains a burning issue. The MDR, which refers to acquired nonsusceptibility to at least one agent in three or more antimicrobial categories, currently constitutes a major obstacle to successful treatment of patients [30]. Based on various studies, the prevalence of MDR UPEC isolates in developing countries is high, varying from 42% (China) through 49.8% (Iran) and reaching an alarming 68% in Pakistan or even 98% in Mexico [31,32,33,34,35,36,37]. Rising rates of antimicrobial resistance result from irrational antibiotic policies, mainly because of inadequate broad spectrum antibiotic empirical therapies prescribed without collecting urine cultures and determining antibiotic susceptibility. MDR bacteria, UPEC included, are responsible for 25 thousand deaths every year in Europe alone, and for 700,000 deaths worldwide [38,39]. 

The phenomenon of antibiotic resistance among bacteria is often attributed exclusively to misuse and overuse of antimicrobial agents. Nevertheless, studies regarding environmental bacteria and human bacterial commensals also revealed that various resistance factors were present within their genomes prior to the commencement of the antibiotic era. The natural presence of antibiotic resistance is referred to as intrinsic antibiotic resistome and occurs in all bacterial species [24,40]. *E. coli* owes its intrinsic resistance mainly to the outer membrane and the expression of efflux pumps [41,42,43]. In contrast, extrinsic antibiotic resistance mainly results from human activity and antibiotic overuse and may be achieved by bacteria in many ways (discussed below).

### 3.1. Bacterial Efflux Pumps

**Figure 3:**
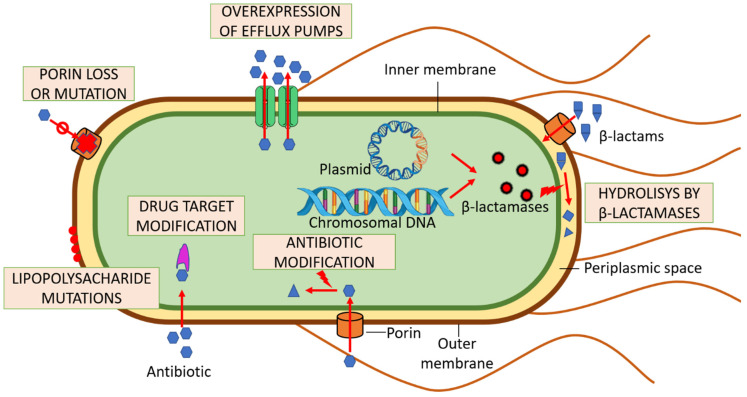
Various mechanisms of multi-drug resistance in *E. coli.* The resistance mechanisms and examples of affected antibiotic agents are: (i) efflux pumps: quinolones, tetracyclines, macrolides; (ii) hydrolysis: β-lactams, macrolides; (iii) antibiotic modification: aminoglycosides, macrolides; (iv) drug target modification: aminoglycosides, quinolones, sulfonamides; (v) lipopolysacharide mutations: colistin, aminoglycosides; (vi) porin loss or mutation: β-lactams, quinolones, tetracyclines.

Antimicrobial resistance can be reached through miscellaneous mechanisms (Figure 3). One of them are bacterial efflux pumps, which can regulate the intracellular environment by removing toxic substances, such as drugs, hence avoiding intracellular accumulation of antimicrobial molecules on their targets. Generally, bacteria contain two-domain half-transporters, which consist of transmembrane domains (TMD) and nucleotide-binding domains (NBD).

There are several efflux systems in *E. coli* associated with drug resistance, which can export drugs and other substrates out of bacterial cells. The seven major efflux pump superfamilies are: (i) the ATP-binding cassette (ABC); (ii) the major facilitator (MFS); (iii) the multidrug and toxic compound extrusion (MATE); (iv) the small multidrug resistance (SMR); (v) the resistance-nodulation-cell division (RND); (vi) the proteobacterial antimicrobial compound efflux (PACE); and (vii) the p-aminobenzoyl-glutamate transporter (AbgT) [44]. ABC transporters are ATP-dependent proteins, while other efflux systems use proton kinetic potential as their driving force.

#### 3.1.1. The ABC Transporters

The ABC transporters are one of the most investigated efflux proteins. Among them, a special place belongs to the lactococcal homodimeric ABC-type protein (LmrA), which is considered as the classic, most-studied MDR pump, capable of transporting various drugs including antimicrobials and chemotherapeutic agents. It mainly removes drugs from the cell membrane, but not from the cytoplasm, and can be inactivated by reserpine or verapamil. P-glycoprotein 1 (P-gp) is a human transporter protein which is homologic to LmrA, sharing its structural and functional features. The activation of MDR pumps is considered as one of the causative factors of ineffectiveness of cancer chemotherapy [45,46,47]. LmrA is also homologous to MsbA, an essential ABC transporter in *E. coli* involved in the trafficking of lipids, including lipid A (endotoxin), which acts as the hydrophobic anchor of lipopolysaccharide (LPS) [48]. MsbA is a homodimeric half-transporter that allows for unidirectional LPS transport. MsbA confers multidrug resistance in *E. coli* and demonstrates overlapping substrate specificities when compared with LmrA. It also displays daunomycin, vinblastine, and Hoechst 33342-stimulated vanadate-sensitive ATPase activity. Interestingly, deletion of MsbA causes membrane disorganization, resulting in cell death [49]. This observation indicates that MsbA might be considered as a potential target of antibacterial therapy, including UPEC [49].

#### 3.1.2. The MacAB–TolC Pump Complex

Another emphasized drug efflux system of *E. coli* that has been reported belongs to the MacAB–TolC pump complex. The system acts as a single drug efflux transporter of macrocyclic lipids, therefore it confers resistance to macrolides. The MacAB–TolC efflux system consists of three main parts: the outer membrane channel (TolC), creating passage for antimicrobials through the outer membrane; the inner membrane transporter, which recognizes drug molecules and activates efflux (AcrB or MacB); and the periplasmatic membrane fusion protein (MPF). Interactions between the TolC and inner member transporter are mediated by the MPF [50].

#### 3.1.3. The AcrAB–TolC and MexAB–OprM Pump Complexes

AcrAB–TolC and MexAB–OprM are other efflux systems essential for effective bacterial colonization of the host as they provide protection against toxic substances. AcrAB–TolC genes and mutations in their efflux repressor AcrR were proposed to cause omeprazole resistance in UPEC [51]. The AcrAB–TolC system is a major player in creating intrinsic resistance to erythromycin and fusidic acid. Moreover, it protects bacterial cells from dyes and detergents. Its importance may be indicated by the fact that it is constitutive among *E. coli* [51]. The AcrAB–TolC system also plays a role in tetracycline resistance along with a variety of other drugs. A strong correlation between AcrA overexpression and ertapenem was recently reported by Chetri et al. [52]. They also observed that the AcrB system is overexpressed when bacteria are exposed to imipenem. Such boosted expression of the mentioned systems is a cause of concern, as it leads to increased non-susceptibility to carbapenems. 

Overall, the *E. coli* genome is predicted to encode 69 ABC transporters. Eleven of them are presumed to be exporters, of which seven (i.e., YddA, YbhFSR, YhiH/YhhJ, YadGH, MdlAB) possibly export drugs. Nevertheless, the bacterial efflux pumps remain largely unexplored, and their role in developing the MDR phenomenon and revealing the full spectrum of substrates needs to be elucidated, as there are limited data regarding not only K1 UPEC, but also K12 *E. coli*.

### 3.2. Enzymatic Decomposition of Antibiotics 

Another crucial mechanism resulting in MDR is the bacteria’s ability to enzymatically decompose antibiotic molecules. A perfect example of this phenomenon is the hydrolytic activity of β-lactamases produced by bacteria, which can inactivate β-lactam antibiotics [53].

#### 3.2.1. β-lactams

β-lactams are widely administered antimicrobial agents that inhibit the biosynthesis of the bacterial cell wall, which prevents the multiplication of subsequent generations of bacteria. They can be divided into four main groups: (i) penicillin derivatives, (ii) cephalosporins, (iii) monobactams, and (iv) carbapenems. The first two are usually first-choice antibiotics in management of UTIs (amoxicillin or cefuroxime axetil are the most commonly prescribed), while the next two groups are reserved to treat patients with life-threatening conditions [54]. BlaTEM, the first group of β-lactamases described in Gram-negative bacteria, along with BlaCTX-M are the most common β-lactamases among *E. coli* detected worldwide. Genes encoding both these groups are mostly transferred by plasmids, but differ in their spectrum of action. *Bla*_TEM_ genes mostly encode β-lactamases that are able to decompose penicillin and first-generation cephalosporins, while *bla*_CTX-M_ encode enzymes which can hydrolyze even third-generation cephalosporins [55]. However, the most worrisome phenomenon is the emergence of broad-spectrum β-lactamases, which confer resistance to all β-lactams, including carbapenems.

#### 3.2.2. Carbapenems

Carbapenems are the last resort antibiotics in treatment of extended spectrum β-lactamase (ESBL)-producing *E. coli*. According to the recent Iraqi study concerning UTI patients, β-lactamase prevalence among *E. coli* is associated with phylogroups [56]. The B2 phylogroup presented the highest antimicrobial resistance with 100% of isolates described as MDR. Moreover, 76.2% of them possessed carbapenemase genes with the most frequent one being *bla*_OXA-48_ (57.8%), followed by *bla*_PER_ (47.3%), while other genes such as *bla*_KPC_, *bla*_VEB_, and *bla*_VIM_ were detected in about 10–15% of UTI isolates. Similar findings were reported by Kuznetsova et al. who noticed that *bla*_OXA_ genes are present in 58.6% of ESBL phenotype-positive isolates. However, the most common β-lactamases belonged to the BlaCTX-M group (79.3%) [57]. Importantly, the B2 phylogroup is characterized as the most virulent type and, as mentioned before, together with the D phylogroup is responsible for the majority of UTIs [56]. Therefore, coexistence of high virulence and UPEC antimicrobial resistance may potentially pose a serious threat to human health [58].

### 3.3. Drug Target Modifications

Bacteria are also able to alternate or protect the target site of antimicrobial drugs, preventing effective treatment. This mechanism can be observed in quinolone resistance. It was thought that quinolone resistance genes (*qnr*) do not exist naturally, as the developed quinolones were fully synthetic antimicrobials. Nevertheless, insusceptibility to quinolones was attributed to either changes in cell wall permeability or point mutations of genes encoding DNA gyrase and DNA topoisomerase IV, which are main quinolone targets [59]. The first chromosomal *qnr* gene, *mfpA*, was reported by Montero et al. in *Mycobacterium smegmatis* [60]. Up until now, *mfpA* homologs have been identified in many organisms, including UPEC (*mcbG* gene) [61].

Qnr proteins are members of the pentapeptide repeat protein family. Qnr proteins competitively attach themselves to the binding site of the DNA gyrase and topoisomerase IV. This prevents fluoroquinolones from forming and stabilizing the gyrase-cleaved DNA-quinolone complex, leading to cell death. Qnr proteins themselves confer low-level quinolone resistance. Additionally, researchers reported that Qnr proteins facilitate the selection of quinolone-resistant mutants; however, the exact molecular mechanism of this phenomenon remains unclear and needs to be elucidated [62,63]. Quinolone resistance among UPEC is mainly plasmid-mediated and is coded by the *qnrA*, *qnrB*, *qnrC*, *qnrD*, and *qnrS* genes [64].

### 3.4. Antibiotic Molecule Modification

Lack of hydrolytic enzyme inactivation by aminoglycoside acetyl transferase enzymes is another mechanism contributing to antibiotic resistance. Proteins taking part in this process in *E. coli* are coded by the *aacA4*, *aadA2*, and *aacC2* genes. The mentioned genes encode acetyltransferases, which catalyze transfer of an acetyl group from acetyl-CoA to a 6′-amino group of aminoglycoside molecules. This confers resistance to antibiotics containing the purpurosamine ring, such as amikacin or kanamycin. Insensitivity to aminoglycosides in UPEC is mostly spread by plasmids, such as pGUE-NDM [36,65,66,67,68]. 

The UPEC antibiotic resistance genes (ARGs), their mechanism of action, and the antimicrobials they affect are summarized in Table 1.

The abovementioned mechanisms do not cover all the possible origins of antibiotic resistance in bacteria. Antibiotic resistance may also originate from random point mutations or can be the result of changes in cell membrane permeability [69,70]. Bacteria may acquire resistance to various antimicrobials by horizontal gene transfer (HGT) and mobile genetic elements (discussed below).

## 4. Mobile Genetic Elements in Antibiotic Resistance

**Figure 4:**
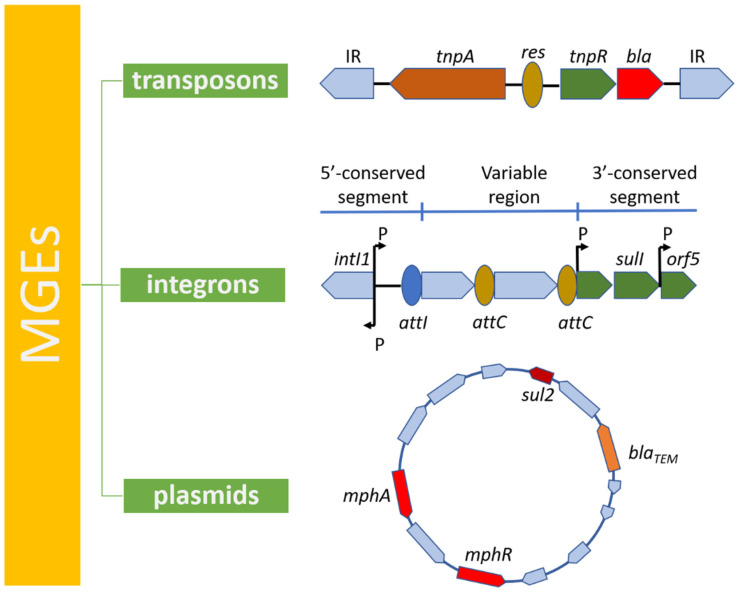
Mobile Genetic Elements (MGEs): transposons, integrons and plasmids are the leading drivers in spreading antimicrobial resistance in many bacteria.

The emergence of new MDR strains that pose a global health threat can be attributed to the circulation and acquisition of already existing ARGs among different populations of bacteria rather than to the emergence of novel resistance genes. Dissemination of ARGs is greatly facilitated by mobile genetic elements (MGEs) which are recognized as the leading drivers in spreading antimicrobial resistance in many bacteria, including UPEC. MGEs are a type of genetic material that can shift within a genome or can be transferred from one strain or replicon to another, allowing the capture, accumulation, and dissemination of resistance genes. MGEs include integrons, transposons, and plasmids [71,72] (Figure 4).

MGEs are found in all organisms and can be identified using various whole-genome alignment systems. One of the examples is the *Mauve* open software package for constructing multiple genome alignments in the presence of large-scale evolutionary events such as inversions and rearrangements [73]. Multiple genome alignments create foundation for research into comparative genomics and the observation of genome-wide evolutionary dynamics. The Mauve tool has its limitations as it is only capable of detecting large insertions. In order to find smaller MGEs, the *breseq* tool can be used [74]. Whole-genome alignment-based methods have their drawbacks, as they are unable to detect insertions of repetitive elements and are dependable on already known MGE sequences. Lately, Durrant et al. developed a bioinformatic toolbox—a MGE finder capable of detecting the MGEs’ genomic sites by using short-read sequencing data, which can overcome the mentioned obstacles [75,76].

### 4.1. Transposons

Transposable elements (TEs) are discrete DNA sequences that can change their position within a genome in almost random pattern. TEs can be assigned to one of two classes based on their mechanism of transposition. Class I transposons (also known as retrotransposons or *copy and paste* TEs) are copied in a two-step process. Firstly, they are transcribed from DNA to RNA, and then the newly synthetized RNA, with the use of reverse transcriptase, is transcribed back to DNA and can then be inserted back into the genome at the new locus. Class II transposons (named *cut and paste* TEs) act in a different way as they do not require an RNA intermediate. The process of transposition in this case is mediated by transposase enzymes, which can bind to specific or any target DNA sequence, depending on their type. The enzyme creates a staggered cut at the target DNA, resulting in formation of sticky ends, then it cuts out the DNA transposon and merges it into the target sequence. The process of transposition can occur autonomously if the transposon itself encodes reverse transcriptase or transposase in Class I and Class II TEs, respectively. Otherwise, presence of another (non-autonomous) TE is required for transposition to happen. TEs are responsible for spreading antibiotic resistance since they may be involved in conferring resistance to multiple classes of antimicrobial drugs, from the commonly used β-lactams to last-resort antibiotics such as colistin [77,78,79]. 

Recently, Carvalho et al. characterized an MDR UPEC BH100 sub-strain. This strain was obtained from the urinary tract of a Brazilian woman in 1974, but was not fully described until now. The usage of the Next Generation Sequencing (NGS) technique allowed assessing the entire sequence of the genome of the strain and it was found that the bacterium harbors two plasmids carrying MDR cassettes: pBH100 and pAp, presenting conjugative and mobilization properties, respectively [80]. The authors indicated transposon Tn*21* as the one likely responsible for the mobilization of the streptomycin resistance gene—*aadA* (encoding for streptomycin 3′-adenylyltransferase). Tn*21*, along with a group of closely linked transposons, forms the Tn*21* family, which is greatly involved in the spreading of antibiotic resistance factors in gram-negative bacteria worldwide [81]. The Tn*21* family carries the integron In1 sequence, encoding a site-specific system for the acquisition and accumulation of multiple antibiotic resistance gene cassettes. Moreover, Tn*21* also carries genes for its own transposition, making it an autonomous TE. 

Carvalho et al. [80] also described the Tn*3* transposon, associated with the *bla*_TEM_ gene, which encodes the β-lactamase TEM-1 type enzyme. Members of the Tn*3* transposon family form a widespread and homogeneous group of bacterial TEs that play a major role in disseminating antibiotic resistance. Tn*3* members use DD (E/D) transposases in the process of replicative transposition in order to create a cointegrate structure, which is later resolved by means of site-specific recombination occurring between specific DNA sequences on both transposon copies. These regions also include promoters, which regulate expression of the recombinase and transposase. Moreover, they also stated that some Tn*3* members encode a type II toxin-antitoxin (TA) system, which improves plasmid maintenance within a bacterial cell. The TA system usually consists of a stable toxin and a labile antitoxin that binds the toxin, therefore preventing it from being lethal for the bacteria [80,82,83,84].

A typical representative of the Tn*3* family consists of three genes: *tnpA, tnpR*, and *bla,* which encode trasposase, resolvase, and β-lactamase, respectively. Recently, Lima-Mendez et al. reported that the members of the Tn*3* family are able to carry a variety of passenger mutations caused by incorporation of nucleic acid fragments into their sequences [84]. Other members of the Tn*3* family are Tn*1546* and Tn*5393*, which carry vancomycin (*vanA*) and streptomycin (*strAB*) resistance, respectively. However, lately there have been no reports of them among UPEC [71]. 

Roy Chowdhury et al. recently described *E. coli* ST*405* as an emerging pathogen of urosepsis. ST*405* presents broad multidrug resistance, as it is unaffected by many antimicrobial agents including ampicillin, azithromycin, kanamycin, streptomycin, trimethoprim, and sulphafurazole [85]. Resistance genes encoding insusceptibility factors to the mentioned antibiotics are: *bla*_TEM-1b_ (β-lactamase), *mphR-mrx-mphA* (macrolide inactivation gene cluster), *aadA5* (aminoglycoside adenyltransferase), *dfrA17* (dihydrofolate reductase), and *sul1* (dihydropteroate synthase). They reside within a novel, mobile transposon named Tn*6242*, the structure of which is composed of four modules. Each module can comprise different antimicrobial resistance genes and is flanked with insertion sequences 26 (IS*26*). The IS are small (<2.5 kilobase long) DNA fragments encoding transposases. IS*26* plays an important role in accumulation and spreading of multiple antibiotic resistance [86]. Tn*6242* is able to generate smaller translocatable units, and a combination of different modules is also remarkable [85,87]. 

TEs also take part in spreading resistance to tetracyclines. Rafaque et al. described an emerging, highly resistant UPEC ST38 O1:H15 strain, which possess various ARGs and MGEs, among which there are two transposons, Tn*2* and Tn*3*, carrying the *tetA gene* [88]. Other Tn*3*-like TEs involved in tetracycline resistance in MDR ST131-O25b-H30 UPEC and causing community-acquired UTIs were also reported by the same authors [89].

Importantly, Tn*6330*, a composite transposon present on both the pHS30-1 plasmid and the chromosome, was highlighted by Li et al. [90]. Tn*6330* was found to be associated with the *mcr-1* gene, which encodes phosphatidylethanolamine transferase. This enzyme is responsible for resistance to colistin, a last-resort antibiotic used in life-threatening infections caused by Gram-negative bacteria [90].

### 4.2. Integrons

Integrons belong to MGEs and are composed of three basic elements: (i) a gene encoding for a site-specific recombinase—integrase (*intI*); (ii) a recombination site (*attI*), which may be associated with various gene cassettes and is recognized by the mentioned integrase; and (iii) a promoter (*Pc*), the role of which is to direct transcription of cassette-encoded genes. It has been discovered that integrase plays a part in recruiting new gene cassettes into integrons, and can also modify those already inserted. Integrons are able to acquire new genes by a reversible cassette-associated recombination process that leads to incorporation, and subsequently expression, of new genetic material in bacteria. This phenomenon is responsible not only for bacterial evolution, as it enables bacteria to adapt to the changing surrounding environment, but also plays a role in acquiring and dissemination of antibiotic resistance, the latter one being a matter of concern in medicine [91,92,93,94]. Integrons can be classified into three main classes based on the *intI* gene. Class-1 integrons are a pivotal driver in spreading antimicrobial resistance genes. They are characterized by considerable diversity and high prevalence, as they have been detected in 22–95% of Gram-negative clinical isolates [95,96,97]. According to Poey and Lavina, class-1 integrons virtually always confer sulfonamide resistance as the already mentioned *sul1* integrates the *IntI*-conserved platform [96]. Furthermore, this class of integrons is also usually associated with cassettes encoding resistance to streptomycin (*aadA* gene) and trimethoprim (*dfrA* gene) [67,98]. This stays in accordance with the recent study performed by Gonzalez-Villalobos et al. on MDR UPEC, where the presence of class-1 integrons was connected with *dfrA5*, *dfrA12*, *dfrA17*, *dfrB4* (trimethoprim/sulfametaxazol), *aadA1*, *aadA2*, *aadA5* (aminoglycosides), and *ereA2* (erythromycin) resistance genes [99]. EreA2 is an integron-encoded erythromycin esterase that is able to hydrolyze erythromycin’s lactone ring. Nevertheless, class-1 integrons have been associated with more than 130 antibiotic resistance factors; therefore, resistance to all classes of antimicrobial agents has been reported [98]. Oliveira-Pinto et al. detected class-1 integrons in 65% of UPEC populations, but only in 11.9% of commensal *E. coli*, which suggests that these integrons may not be an indigenous part of *E. coli* [98]. This is consistent with the data from Gonzalez-Villalobos et al., who detected class-1 integrons in 63.9% of UPEC pathogenic phylogroups (B2 and D), and not in commensal ones. Similar data were reported by Poey et al., where a strong association between class-1 integrons and the phylogenetic group D was observed [96,99]. 

Class-2 integrons are less common, but also play a substantial role in the spread of resistance genes. They are usually found in strains from phylogroups A, B1, and B2 [96]. Shams et al. studied drug-resistant *E. coli* obtained from various clinical specimens and observed an association between the presence of integrons and insensitivity to various antimicrobial agents. They discovered that presence of class-1 integrons is related to resistance to ceftriaxone, ceftazidime, gentamicin, and nalidixic acid, whereas class-2 integrons were associated with insensitivity to imipenem, nalidixic acid, and co-trimoxazole. 

It is worth mentioning that there is concern about high co-prevalence of class I and II integrons with ESBL and MDR in quinolone-resistant isolates [95]. The described association between class-1 integrons and antimicrobial resistance is partially consistent with more recent reports. For example, Mirnezami et al. studied UPEC strains and showed a statistically significant relationship between presence of class-1 integrons and resistance to gentamicin, ciprofloxacin, co-trimoxazole, ampicillin, and nalidixic acid. However, no significant association with ceftazidime resistance was observed [100]. 

Class-3 comprises the least frequently-observed integrons and are less often associated with antibiotic resistance. Until now, only one study by Xicohtencatl-Cortes et al. described class-3 integrons associated with MDR in the UPEC B2 phylogroup [101]. 

### 4.3. Plasmids

Plasmids are also considered as members of MGEs and are defined as small, extrachromosomal DNA circular molecules that possess the ability to replicate independently. They are most common among bacteria, however, plasmids may also be created artificially, and, therefore, play an important role in genetic engineering as vectors. Plasmids may be classified according to their function into five main classes: (i) virulence, (ii) resistance (R), (iii) fertility (F), (iv) degradative, and (v) colicin (Col) plasmids. The latter one harbors genes encoding for proteins toxic to other bacteria (bacteriocins). Nevertheless, the most used plasmid classification method currently is incompatibility (Inc) typing [102].

Most plasmids are double-stranded DNA (dsDNA), but some consist of single-stranded DNA (ssDNA) or double-stranded RNA (dsRNA). Plasmids play a substantial role in spread of antibiotic resistance worldwide, as some of them carry antimicrobial resistance genes that can be transferred between bacteria. Based on the Inc plasmid typing scheme, until now, 28 types of antibiotic resistance plasmids have been described. The IncF type plasmids are the most common in human and animal sources, and are mainly found in *E. coli*, including UPEC [103]. Unlike most plasmids, the IncF group has the ability to encode several replicons. IncF plasmids can carry a variety of resistance genes: ESBL, genes encoding carbapenemases, aminoglycoside-modifying enzymes, and plasmid-mediated quinolone resistance (PMQR) genes (discussed later). Importantly, IncFs are most often associated with ESBL resistance, as they spread *bla*_CTX-M_, *bla*_TEM-1_, and *bla*_NDM_ genes [102].

Resistance plasmids can also be assigned one of two groups based on their mobility: narrow host range plasmids, which are usually transferred between the same species, and broad host range plasmids that can easily be harbored by different bacteria. The second group is associated with dissemination of MDR [104]. 

A recent study by Muriuki et al. from Kenya indicated that the main mechanism of resistance to β-lactam antibiotics in UPEC is the production of ESBLs and plasmid-mediated β -lactamases (pAmpCs) [105]. The occurrence of ESBLs depends on the geographical region and positively correlates with the overuse of antimicrobial agents. Prevalence rates in developing countries vary from 5% to 44.3%. AmpC β-lactamases confer insensitivity to penicillins, cephems, and monobactams, but the bacteria remain susceptible to carbapenems. Addition of β-lactamase inhibitors clavulanate, sulbactam, or tazobactam does not overcome antibiotic resistance in these cases [105]. PAmpCs originate from chromosomally-encoded AmpCs of multiple genres among the *Enterobacteriaceae* family, do not create a homogenous group, and the most frequent one is the CMY type.

Another worrying phenomenon is the growing resistance of bacteria to fluoroquinolones. According to Sadeghi et al. and Ramirez-Castillo et al., who examined UPEC strains obtained from kidney transplant patients (KTPs) and non-KTPs, plasmid-mediated quinolone resistance (PMQR) is increasingly identified [36,106]. Quinolone resistance is caused by various mechanisms, with PMQR being one of the main players. PMQR is associated with the *qnrA*, *qnrB*, *qnrC*, *qnrD*, and *qnrS* genes, which encode proteins that protect quinolone targets—gyrase and topoisomerase IV—from being affected by this group of antimicrobial agents. 

PMQR is also associated with genes encoding efflux pumps *qepA* and *qqxAB*. QepA was reported to be linked with decreased susceptibility, and 8- to 32-fold increase of minimum inhibitory concentration (MIC) values of hydrophilic quinolones, such as norfloxacin, ciprofloxacin, and enrofloxacin [107]. QqxAB was characterized by a wider substrate specificity, including chloramphenicol, trimethoprim, and quinolones (ciprofloxacin, norfloxacin, and nalidixic acid) [108]. Even though PMQR is responsible for inducing only low-level resistance to quinolones, it could spread horizontally among other bacteria and elicit the selection of resistant strains, facilitating the acquisition of other quinolone-resistance mechanisms, thus resulting in high-level resistance. Acquiring quinolone resistance is especially dangerous for KTPs, as quinolones are used in UTI prophylaxis.

Moreover, PMQR strains often co-harbor genes encoding ESBL proteins, resulting in creation and selection of MDR UPEC [36,106,109]. Co-resistance to β-lactams and quinolones was also investigated in the study by Basu et al. [110]; 85% of strains collected from UTI patients were classified as MDR, where 86% of them were resistant to ciprofloxacin —a first-line drug in UPEC treatment. PMQR was detected in 50% of quinolone-resistant strains; moreover, all the isolates co-harbored at least one gene encoding β-lactamase, where *bla*_TEM_ was the most common. This study also indicated that resistance genes can be transferred by conjugation upon ciprofloxacin selection.

Another example of plasmid-mediated antibiotic resistance in UPEC is azithromycin-resistance associated with presence of the *mphA* gene reported in cohorts of UTI patients previously not exposed to antibiotics. The spread of macrolide-resistant *E. coli* in the environment may become a threat to the treatment of other diseases, such as diarrheas caused by *Salmonella* and *Shigella* spp., as plasmids can be easily transferred [111].

## 5. Obstacles in UTI Treatment

The rapid dissemination of MDR UPEC observed worldwide could partially be attributed to gene migration. However, the mainspring of increasing antibiotic resistance is overuse and unreasonable antimicrobial policies. Frequent antibiotic exposure provides ideal conditions for selection of multi-drug resistant strains, which can later transfer resistance genes to other non-pathogenic or pathogenic bacteria present in the environment. UTI treatment may soon pose a challenge, as the situation is made worse by the lack of new antimicrobials. Recently, only three major pharmaceutical companies have been conducting research on developing new antibiotics [38,112,113].

It is observed that antimicrobial agents are less effective at killing intracellularly-localized bacteria; hence, IBC-forming cells are more likely to survive the antibiotic course and re-infect other bladder cells [114,115,116]. Therefore, increasing interest is now paid to inhibitors, which are considered as a potential tool to fight UPEC IBC’s. An example of such a compound is mannoside, capable of inhibiting FimH lectin, which plays a role in forming IBCs. According to Cusumano et al., they seem promising for patients with recurrent UTIs. However, until now, no FimH inhibitors have undergone full clinical trials [117]. Another recent trial addressed inhibitors (ZINC000104153710; ZINC000000217308) of universal stress protein A (UspA) as they present the capability to bind to the UspA molecule, and affect IBCs [118].

One of the mechanisms engaged in formation of UPEC IBCs, manifesting morphological flexibility, is filamentation. It is believed that development of filamentous cells is one of the many bacterial strategies implemented to avoid host immune responses, as it provides protection against phagocytosis. It is also a natural way to increase the gene copy number and it is a hallmark of IBCs. The process of filamentation is stimulated by stress factors, including the activated host immune system, but it can also be induced by antimicrobials. β-lactams inhibit penicillin-binding protein-3 (PBP-3), which takes part in septa formation, therefore facilitating filamentation. 

The process of filamentation is controlled by the SOS response system. SOS is common and conserved in various bacteria; it is recruited in case of significant DNA damage and induces multiple proteins to protect integrity of DNA. The SOS pathway plays an essential role in increasing the resistance to stress and, therefore, to antibiotics. However, it also stimulates error-prone polymerases, allowing for enhanced survival, but also increasing mutagenesis at the same time [119,120]. The SOS response and its association with antibiotic resistance have recently been highlighted [121,122]. It has been proven that antimicrobials such as trimethoprim, fluoroquinolones, β-lactams, and aminoglycosides are potent activators of the SOS system by interfering with DNA replication or cell wall synthesis [123]. Activation of the SOS pathway results in increased mutational frequency, facilitating the rise of mutations in genes encoding antimicrobial targets, such as *gyrA* (DNA gyrase subunit A) in case of fluoroquinolones, making them ineffective. Antibiotics like quinolones, β-lactams, and aminoglycosides can kill bacteria by inducing ROS formation, which damages DNA. In response, the SOS pathway is recruited as DinF, a DNA-damage-inducible SOS response protein is able to decrease intracellular ROS levels, preventing bacteria from oxidative injuries and cellular death. The SOS system is also associated with HGT and MGEs, especially integrons, as it can induce the recombination of gene cassettes by controlling expression of the *intI* gene [124].

The highlighted close link between antibiotic resistance and the SOS system may be a potential target of new drugs—SOS modulators, which might be a tool to fight MDR bacteria [122]. Inhibition of the SOS pathway can be achieved either by LexA or RecA inactivation and results in a reduction in MIC for some antibiotics. RecA can be inhibited by Suramin, N6-ADP, curcumin, or zinc, while LexA only has a few potential inhibitors—small boron-containing compounds [125]. Crane et al. discovered that SOS-inducing drugs, such as ciprofloxacin or zidovudine, cause increased resistance to other antibiotics—rifampin, minocycline, and fosfomycin—by triggering hypermutations. Zinc, in vivo, was able to inhibit this SOS-induced appearance of antimicrobial resistance [122,126]. A similar effect was also described for N-acetylcysteine [127]. Moreover, Recacha et al. found that suppression of the SOS system can even reverse antibiotic resistance among highly-resistant quinolone strains [128]. The potential ability of SOS inhibitors to block the emergence of antimicrobial resistance or increase antibiotic effectiveness in vivo requires further research.

## 6. Conclusions

Mobile genetic elements play a major role in dissemination of antimicrobial resistance genes among various bacteria. The growing antibiotic resistance of pathogenic bugs resulting from overly prescribed antibiotics may soon pose an enormous challenge. In the absence of new classes of antimicrobial drugs and the bacteria’s ability to rapidly adapt to various stress conditions with the use of the SOS pathway, we should seek alternative methods of treatment of bacterial infections. SOS inhibitors seem a promising alternative, as they have not only been proven to be effective, but also the pathway they target is conserved in many different bacteria species. Nonetheless, SOS modulators require further study and for now strategies should focus on implementing rational antibiotic policies.

## Figures and Tables

**Table 1:** The UPEC antibiotic resistance genes—ARGs, their mechanisms of action, and antimicrobials they affect.

Antimicrobial Class/Agent	ARG	Mechanism of Resistance
β-lactams	*bla*_TEM_	hydrolysis of the antibiotic molecule
	*bla*_OXA_	
	*bla*_CTX-M_	
	*bla*_SHV_	
	*bla*_VEB_	
	*bla*_PER_	
	*bla*_KPC_	
	*bla*_VIM_	
	*ampC*	
Aminoglycosides	*aadA*	antibiotic molecule modification (adenylyltransferase)
	*armA*	drug target modification (methylase)
	*rmtB*	
	*aaC(3)—IIa*	antibiotic molecule modification (acetyltransferase)
	*aacA2*	
	*aacA4*	
Quinolones	*qnrA*	drug target modification
	*qnrB*	
	*qnrC*	
	*qnrD*	
	*qnrS*	
	*mfpA*	
	*qepA*	efflux pump
	*oqxAB*	
Tetracyclines	*tet(a*)	efflux pump
	*tet(b*)	
Sulfonamides	*dfrA1*	drug target modification
	*sul1*	
	*sul2*	
Macrolides	*ere(2*)	hydrolysis of the antibiotic molecule
	*acrB*	efflux pump
	*acrA*	
	*macB*	
	*mph(A*)	Antibiotic molecule modification (phosphotransferase)
Vancomycin	*vanA*	drug target modification
Colistin	*mcr-1*	drug target modification (phosphatidylethanolamine transferase)

## Abbreviation

ABC	ATP-binding cassette
CNF-1	Cytotoxic Necrotizing Factor type I
DAEC	Diffusely adherent *Escherichia coli*
dsDNA	Double-stranded DNA
dsRNA	Double-stranded RNA
EAggEC	Enteroaggregative *Escherichia coli*
EHEC	Enterohaemorrhagic *Escherichia coli*
EIEC	Enteroinvasive *Escherichia coli*
EPEC	Enteropathogenic *Escherichia coli*
ESBL	Extended spectrum β-lactamase
ETEC	Enterotoxigenic *Escherichia coli*
ExPEC	Extraintestinal *Escherichia coli*
HGT	Horizontal gene transfer
IBC	Intracellular bacterial communities
IS	Insertion sequence
KTP	Kindey transplant patients
MATE	Multidrug and toxic compound extrusion
MDR	Multi drug resistant
MGE	Mobile genetic element
MIC	Minimum inhibitory concentration
MPF	Membrane fusion protein
NBD	Nucleotide-binding domain
NGS	Next Generation Sequencing
NMEC	Neonatal Meningitis *Escherichia coli*
PMQR	Plasmid-mediated quinolone resistance
Qnr	Quinolone resistance
ROS	Reactive oxygen species
SMR	Small multidrug resistance
ssDNA	Single-stranded DNA
TE	Transposable element
TMD	Transmembrane domain
UPEC	Uropathogenic *Escherichia coli*
UTI	Urinary tract infection
